# Efficacy of dupilumab for the treatment of severe skin disease in cytotoxic T lymphocyte antigen-4 insufficiency: A role of type 2 inflammation?

**DOI:** 10.1016/j.jacig.2022.08.004

**Published:** 2022-09-22

**Authors:** L. Karla Arruda, Daniel L. Cordeiro, Sarah S. Langer, Marcel Koenigham-Santos, Rodrigo T. Calado, Marina M. Dias, Leonardo R. Anhesini, João Bosco Oliveira, Bodo Grimbacher, Mariana P.L. Ferriani

**Affiliations:** a Department of Medicine, Ribeirão Preto Medical School, University of São Paulo, São Paulo, Brazil; b Department of Pediatrics, Ribeirão Preto Medical School, University of São Paulo, São Paulo, Brazil; c Department of Medical Imaging, Hematology, and Oncology, Ribeirão Preto Medical School, University of São Paulo, São Paulo, Brazil; d Intensive Care Unit, Hospital São Paulo, São Paulo, Brazil; e Hospital Israelita Albert Einstein, São Paulo, Brazil; f Institute for Immunodeficiency, Center for Chronic Immunodeficiency, Medical Center, Faculty of Medicine, Albert-Ludwigs-University of Freiburg, Freiburg, Germany; g Clinic of Rheumatology and Clinical Immunology, Center for Chronic Immunodeficiency, Medical Center, Faculty of Medicine, Albert-Ludwigs-University of Freiburg, Freiburg, Germany; h German Center for Infection Research, Satellite Center Freiburg, Freiburg, Germany; i Centre for Integrative Biological Signalling Studies, Albert-Ludwigs University, Freiburg, Germany; j RESIST–Cluster of Excellence 2155 to Hanover Medical School, Satellite Center Freiburg, Freiburg, Germany

**Keywords:** Dermatitis, cytotoxic T-lymphocyte antigen-4 insufficiency, dupilumab, abatacept, inborn errors of immunity

## Abstract

We report on the successful treatment of a severe, recalcitrant dermatitis caused by CTLA-4 insufficiency with dupilumab, raising the possibility of a role of type 2 immunity in clinical conditions associated with CTLA-4 insufficiency.

Cytotoxic T lymphocyte antigen-4 (CTLA-4) is a crucial immune checkpoint that is constitutively expressed in regulatory T cells and upregulated on activated T cells.^1^^,^^2^ Heterozygous pathogenic variants in the *CTLA4* gene cause an immune dysregulation syndrome designated as CTLA-4 insufficiency.^1^^,^^2^ Patients with CTLA-4 insufficiency present heterogeneous clinical manifestations, including hypogammaglobulinemia, cytopenias, autoimmunity, hepatosplenomegaly, enteropathy, and lymphocytic infiltrations of nonlymphoid organs. Cutaneous manifestations, mainly atopic dermatitis, may occur in up to 56% of the patients.^3^^,^^4^ Diagnosis requires genetic testing, as well as a CTLA-4–specific functional test when a novel variant is found.^1^ Treatment includes targeted therapies such as the CTLA-4 fusion proteins abatacept and belatacept, the mechanistic target of rapamycin inhibitor sirolimus, immunoglobulin replacement, corticosteroids, and immunosuppressant agents.^2^^,^^3^ Hematopoietic stem cell transplantation may be considered in selected patients.^3^

A 20-year-old female college student with severe skin disease came to our allergy and immunology service for evaluation in August 2020. Her symptoms started in the first year of life with mild flexural dermatitis. At the age of 10 years, she presented with worsening of the dermatitis with lichenified, infiltrating lesions in her legs, arms, and trunk along with severe itching and pain (Fig 1, *A-C*). She reported contact dermatitis with jewelry and nail polish, and patch testing revealed positive results in response to nickel sulfate, cobalt dichloride, propylene glycol, and *p*-tert-butylphenol, which she has avoided for several years. She showed poor response to topical steroids and emollients, and she had received repeated courses of oral corticosteroids and antibiotics for dermatitis flares.

**Fig 1:**
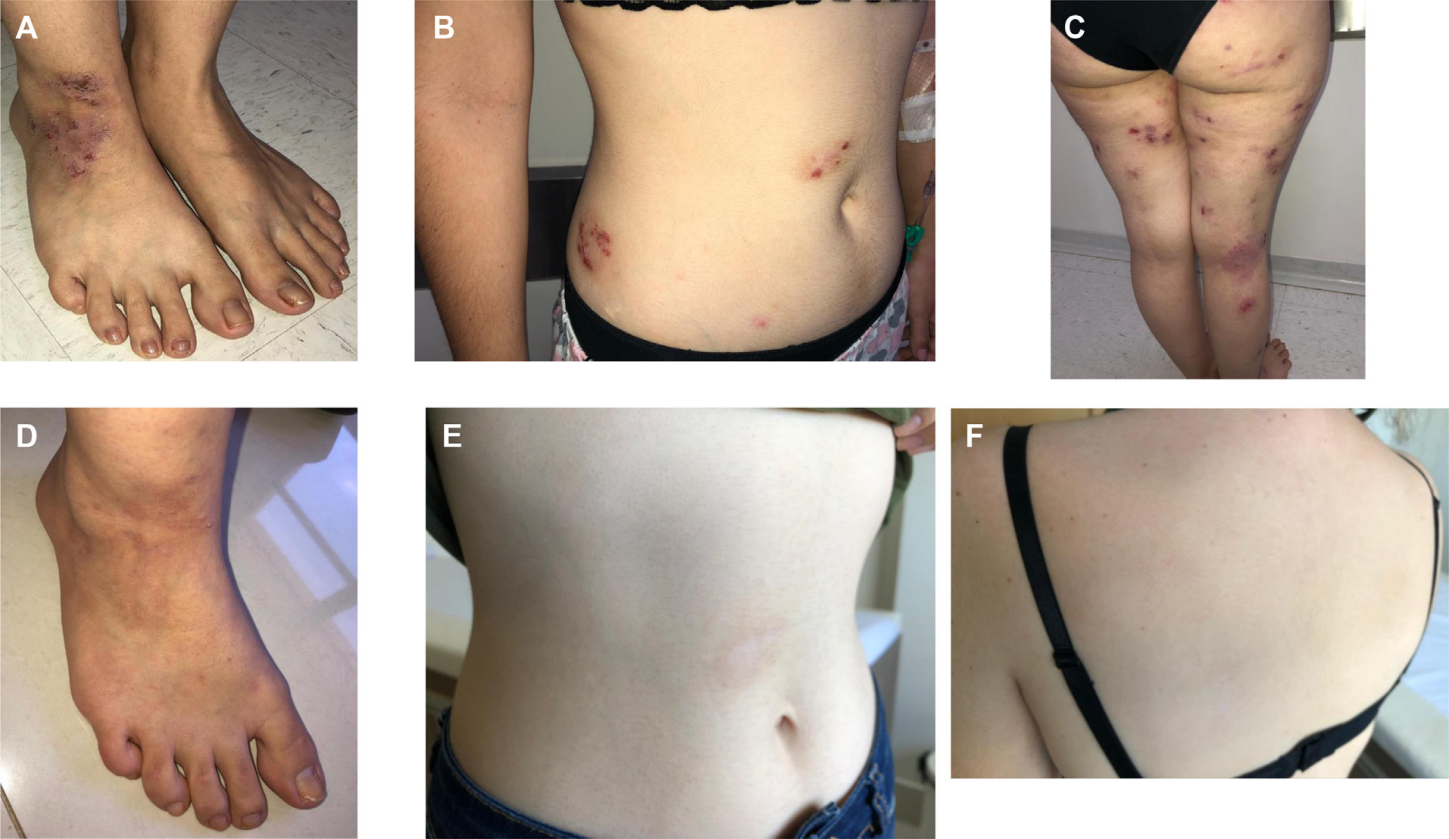
Clinical presentation of the patient with severe skin disease at first consultation (**A-C**) and after 9 months of therapy with dupilumab (**D-F**). Informed consent was obtained from the patient to publish this case report along with the visual elements.

A skin culture with *Staphylococcus aureus* was obtained, and her biopsy results were consistent with atopic dermatitis, showing parakeratosis permeated with leukocytes, spongiosis, and lymphocytic infiltrate in the dermis. Total IgE was undetectable (<1 IU/mL), and no specific IgE to food and inhalant allergens was detected. The patient denied rhinitis or asthma. She reported 2 relevant infectious episodes in her history. In 2018, she was admitted to the hospital for recurrent fever, abdominal pain, and lymphadenopathy. Lymph node and bone marrow biopsies showed inconclusive results. She received broad-spectrum intravenous antibiotics and recovered. In January 2020, after surgery for wisdom teeth removal, she developed a severe local infection spreading to the ears, leading to partial hearing impairment. Her 44-year-old mother was diagnosed with acquired severe aplastic anemia by the age of 24 years and subsequently developed autoimmune hemolytic anemia and hypothyroidism. Her father is 45 years old and healthy.

Further investigation revealed serum immunoglobulin levels that were all below the third percentile.^5^ Her leukocyte and lymphocyte counts were low; in particular, her CD19^+^ B lymphocyte level was low at 10 cells/μL (normal range = 110-618 cells/μL) (see Table E1 in the Online Repository at www.jaci-global.org). She was diagnosed with common variable immunodeficiency, and intravenous immunoglobulin replacement at 400 to 500 mg/kg every 4 weeks was initiated in October 2020; however, no improvement of her skin lesions was observed. Dupilumab, a human mAb directed to the α-chain of the IL-4 receptor, was initiated in January 2021 at a loading dose of 600 mg subcutaneously, followed by 300 mg subcutaneously every 2 weeks.^6^^,^^7^ The patient had a major improvement of her dermatitis (Fig 1, *C-E*), perceived after 2 months of therapy. Her Scoring Atopic Dermatitis score decreased from 80.7 to 25.5; her Atopic Dermatitis Control Test score decreased from 22 to 3; and her Dermatology Life Quality Index decreased from 27 to 1 at 12 months after initiation of treatment with dupilumab versus at baseline.

In April 2021, she was enrolled in the Rare Genomes project. Whole genome sequencing (Illumina NovaSeq 6000, Illumina, San Diego, Calif) revealed a heterozygous, previously published,^3^ pathogenic variant in *CTLA4* (NM_005214.5) (c.410C>T, p.Pro137Leu), allowing for the diagnosis of CTLA-4 insufficiency. The c.410C>T variant was also detected in the patient’s mother by Sanger sequencing.^3^ Investigation by chest computed tomography showed multiple inflammatory pulmonary nodules (see Fig E1 in the Online Repository at www.jaci-global.org), which might represent a manifestation of granulomatous-lymphocytic interstitial lung disease. Mildly enlarged lymph nodes were identified in the mediastinum, without calcification or necrosis, as well as mild splenomegaly. Thickening of the gallbladder wall was observed on abdominal ultrasonography; however, the patient's condition remained well controlled, with no respiratory or abdominal symptoms, and treatment with dupilumab and intravenous immunoglobulin replacement was maintained.

In August 2021 the patient presented with a urinary tract infection that was followed by severe abdominal pain, nausea, vomiting and diarrhea, and weight loss of 15 kg in 3 weeks. A gluten- and lactose-free diet and therapy with oral budesonide, 9 mg per day, were not helpful, and she was admitted to the hospital. An abdominal computed tomography scan showed multiple mesenteric and retroperitoneal enlarged lymph nodes, hepatomegaly, splenomegaly, and thickening of the gallbladder wall (Fig 2). Endoscopy revealed atrophy of duodenal folds, and biopsy showed mild gastritis and chronic nonspecific duodenitis with a decreased villous-to-crypt ratio at 1:1, and up to 18 intraepithelial lymphocytes per 100 enterocytes, which was suggestive of celiac disease. A colonoscopy revealed mild inflammatory infiltrate in the patient's rectum and anal canal. Because of a refractory course, parenteral nutrition was started. She developed pancytopenia: her hemoglobin level dropped to 8.8 g/L, her leukocyte count decreased to 940/μL, her lymphocyte count fell to 301/μL, and her platelet count dropped to 31,000/μL. Her bone marrow was normocellular with mild hyperplasia of megakaryocytes and micromegakaryocytes. Her mesenteric lymph nodes showed a marked inflammatory infiltrate, mainly with lymphocytes. Her soluble IL-2 receptor levels were elevated, at 5530 pg/mL (normal range = 532-1891 pg/mL). On November 9, 2021, the patient began taking abatacept, a fusion protein of the CTLA-4 extracellular domain bound to the Fc fragment of human IgG_1_, with a loading dose of 500 mg administered intravenously, followed by weekly subcutaneous injections of 125 mg. Following the first dose, the patient had marked improvement of her symptoms. Her hemoglobin level went up to 11.2 g/dL, her leukocyte count rose to 4760/μL, her lymphocyte count increased to 476/μL, and her platelet count rose to 89,000/μL. On November 21, 2021, she was discharged from hospital and is doing well. Treatment with dupilumab was maintained and is being continuined in addition to therapy with abatacept and intravenous immunoglobulin replacement.

**Fig 2:**
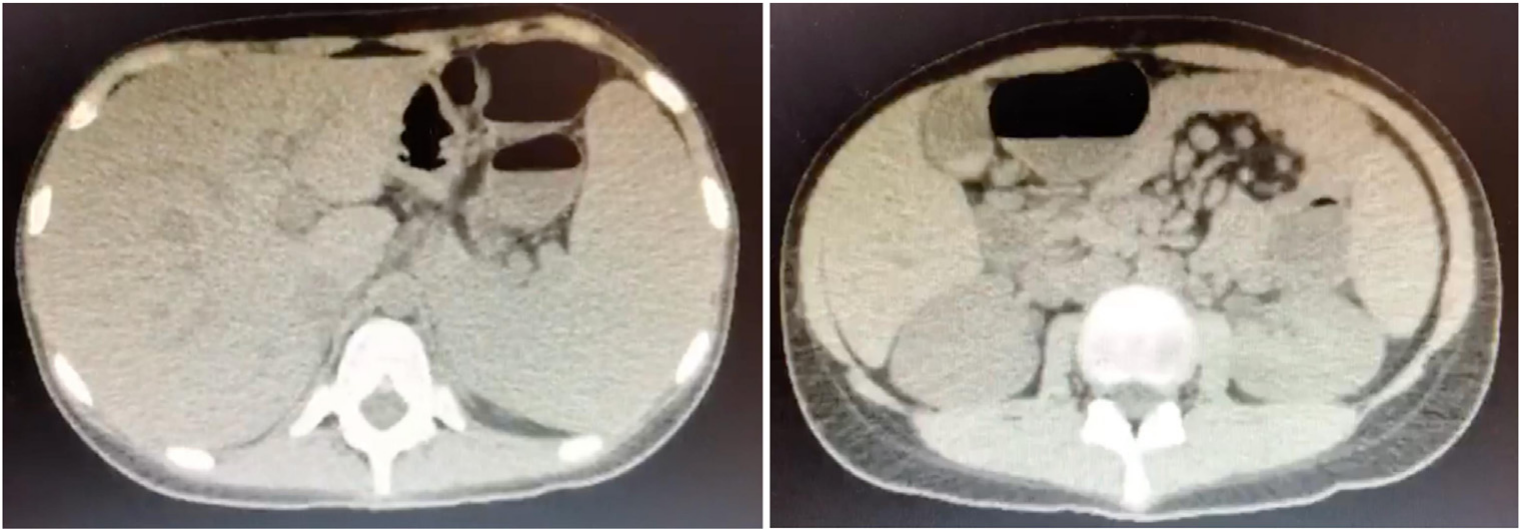
Axial abdominal computed tomography images showing hepatosplenomegaly and enlarged mesenteric and retroperitoneal lymph nodes. Computed tomography images obtained in August 2021 for investigation of severe gastrointestinal symptoms.

Our case report highlights the possibility of an initial presentation of CTLA-4 insufficiency with severe skin disease.^4^ One important question is whether our patient really presented with atopic dermatitis in the absence of an elevated total IgE level or increased blood eosinophil count. Clinically, her treating dermatologists have consistently agreed on a diagnosis of atopic dermatitis; her contact dermatitis were addressed and well controlled; and her skin biopsy results excluded an alternative diagnosis such as cutaneous lymphoma, autoimmune disease, vasculitis, or specific infection. It would have been useful to assess additional potential biomarkers, identified in nonlesional and lesional skin, and in the blood of patients with atopic dermatitis, including IL-13, IL-22, chemokine (C-C motif) ligand 27/cutaneous T-cell–attracting chemokine CCL27/CTACK (upregulated in atopic dermatitis but downregulated in psoriasis), inducible nitric oxidase synthase 2 (NOS2) (upregulated in psoriasis but downregulated in atopic dermatitis), and CCL17/ thymus and activation-regulated chemokine (TARC)^5^; however, these biomarkers are not yet readily available in clinical practice. Ultimately, dupilumab was very effective and safe in our patient, which is strongly suggesting an underlying type 2 inflammation associated with her skin disease.

An undetectable serum IgE level was certainly a matter of concern in our patient. Lawrence et al reported that an undetectable serum IgE level (< 2 IU/mL) occurs in only 3.3% of the general population versus in 75.6% of patients with common variable immunodeficiency.^6^ Their data suggest that an IgE level below the lower limit of detection could prompt investigation of a primary humoral immunodeficiency by screening for suggestive symptoms, including recurrent infections and autoimmunity and measurement of other serum immunoglobulin levels. Therefore, it is possible that had the absense of serum IgE been recognized, the diagnosis of a primary immunodeficiency disease in our patient could have been made earlier, emphasizing the importance of obtaining total IgE measurements and a careful clinical history in patients with atopic dermatitis.

Therapy with dupilumab was considered after failure of topical treatments and intravenous immunoglobulin replacement for hypogammaglobulinemia in ameliorating the patient’s severe skin disease, albeit before the diagnosis of CTLA-4 insufficiency. Considering the diagnosis of probable atopic dermatitis, other options for systemic treatment would have included long-term oral corticosteroids, cyclosporin A, or methotrexate, which may have added to the patient’s immune deficiency. The rational for trying dupilumab was to act on a potential cellular type 2 response by inhibiting IL-4 and IL-13. The proven efficacy of dupilumab in atopic dermatitis and other type 2 diseases not necessarily associated with elevation of IgE level, including chronic rhinosinusitis with nasal polyps and eosinophilic esophagitis, and its favorable safety profile, were important considerations in our decision to institute dupilumab therapy, which turned out to be very effective for our patient.^7^

An increased risk of cancer is a concern among patients with CTLA-4 insufficiency. In the cohort of 131 patients with CTLA-4 insufficiency, 13% of the affected *CTLA4* variant carriers had a malignant cell growth, most frequently EBV-associated lymphomas and gastric cancer.^8^ In an animal model system, induced CTLA-4 insufficiency initiated *de novo* tumorigenesis in the mouse stomach, which progressed to gastric adenocarcinoma over time.^9^ Interestingly, deficiency of IL-4, IL-13 or IL-4 receptor-α, but not of IFN-γ or IL-17A, suppressed tumorigenesis in these mice, suggesting that type 2 immunity is an important player in CTLA-4 insufficiency.^9^

Abatacept has previously shown beneficial effects in some patients with CTLA-4-insufficiency presenting enteropathy, lymphoproliferation, and autoimmune cytopenias,^2^^,^^3^ and in our patient, abatacept was highly effective, leading to fast and sustained improvement of her systemic symptoms. However, the question of whether abatacept without dupilumab would be sufficient to keep her skin symptoms well controlled, and therefore, whether dupilumab should be discontinued, is currently a management challenge to our group. Egg et al have reported on 1 patient without detectable T_H_17 cell infiltration who did not show improvement of the skin condition with abatacept.^2^ Several other issues remain, including the long-term efficacy and safety of abatacept treatment and whether our patient would be a candidate for a successful human stem cell transplantation. We hope that the forthcoming results of prospective studies such as the ABACHAI trial (Safety and Efficacy of abatacept (subcutaneously) in patients with CTLA-4 insufficiency or LRBA deficiency [EudraCT identifier 2019-000972-40]) will shed light on these difficult clinical decisions. In the meantime, we chose to maintain therapy with dupilumab in our patient in conjunction with abatacept and intravenous immunoglobulin replacement therapy.

In conclusion, the patient reported here had severe skin disease, albeit not associated with obvious IgE response. Her skin disease improved coincidently with therapy with dupilumab, but she exhibited findings that led to the diagnosis of a primary immune deficiency. Ultimately, the diagnosis of CTLA-4 insufficiency was made and molecularly driven precision treatment was initiated when she presented with severe systemic symptoms. This case highlights the need for vigilance for primary immune deficiency in persons with severe dermatitis. In addition, the case also highlights the potential for overexpression of type 2–driven inflammation without overt allergen-specific IgE in patients with dermatitis. This relationship requires ongoing surveillance.

## Supplementary data

Supplementary Figure1

Supplementary Table1
