# Supplementary figures and images for: Efficacy of dupilumab for the treatment of severe skin disease in cytotoxic T lymphocyte antigen-4 insufficiency: A role of type 2 inflammation?
Source: J Allergy Clin Immunol Glob. 2022 Sep 22;2(1):114–7. doi: 10.1016/j.jacig.2022.08.004 (PMC10509893; doi:10.1016/j.jacig.2022.08.004)

## Slide 1
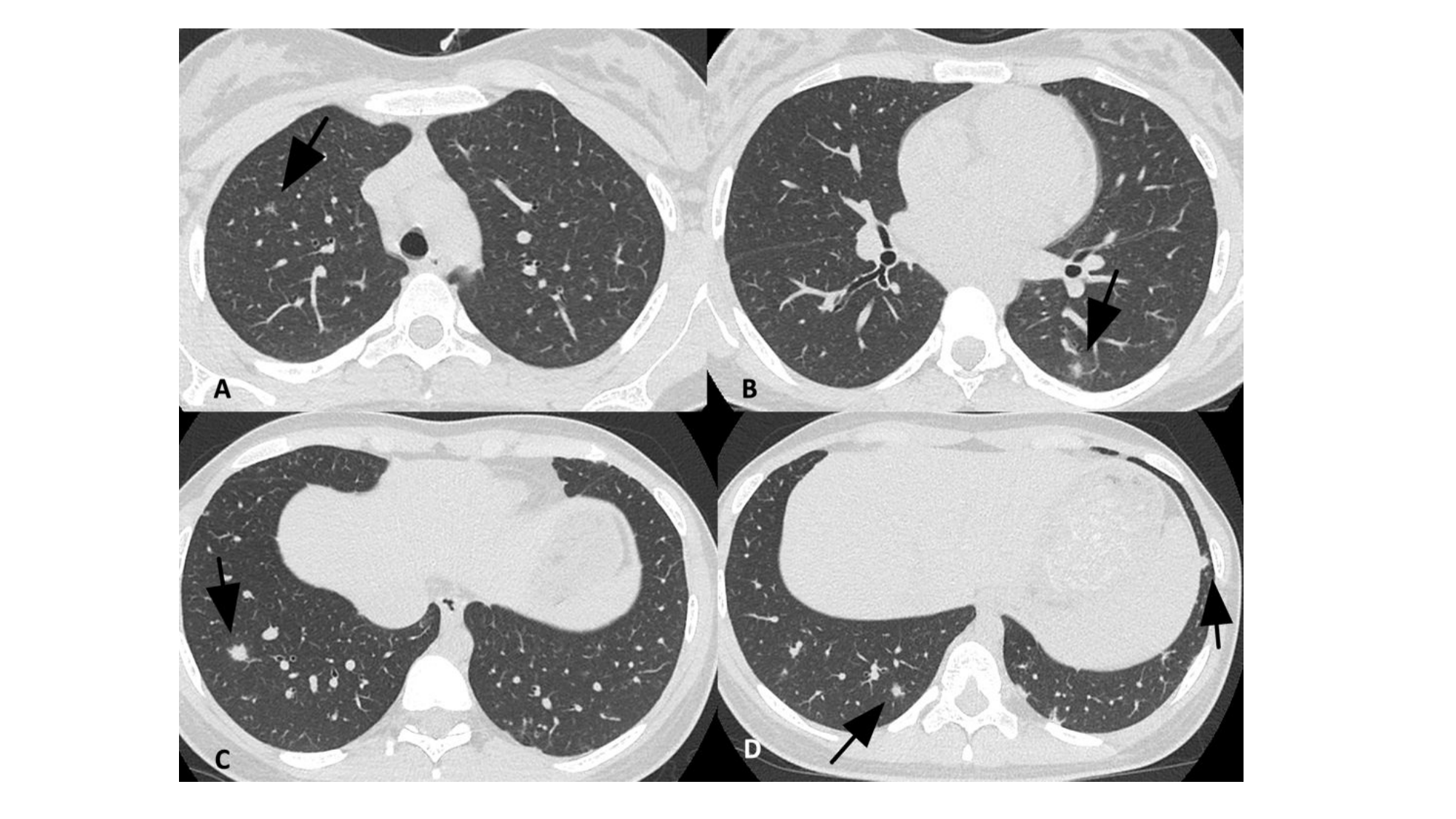

Supplement: Supplementary Figure1 [file mmc1.pptx]
