# Supplementary material for: Efficacy of dupilumab for the treatment of severe skin disease in cytotoxic T lymphocyte antigen-4 insufficiency: A role of type 2 inflammation?
Source: J Allergy Clin Immunol Glob. 2022 Sep 22;2(1):114–7. doi: 10.1016/j.jacig.2022.08.004 (PMC10509893; doi:10.1016/j.jacig.2022.08.004)
Supplement: Supplementary Table1 [file mmc2.docx]

| Parameter^+^ | | Patient’s measurement | Normal reference values |
| --- | --- | --- | --- |
| Hemoglobin (g/L) | | 12.9 g/L | 12-16 g/L |
| Leukocytes | | 2,732/µL | 3,500-10,500/µL |
|  | Neutrophils (%) | 1,490/µL (57%) | 2,500-7,000/µL (54-67%) |
|  | Eosinophils (%) | 297/µL (11%) | < 300/µL |
|  | Lymphocytes (%) | 564/µL (20.9%) | 900-3,500/µL (20-35%) |
|  | Monocytes (%) | 338/µL (12.5%) | 180-1,200/µL (3-14%) |
|  | Basophils (%) | 11/µL (0.4%) | 0-100/µL (0-1%) |
| Platelets | | 141,000/µL | 130,000-450,000/µL |
| Serum immunoglobulin levels, mg/dL^#^ | |  |  |
|  | IgG | 146 mg/dL | 620-1,420 mg/dL (< 3^rd^ percentile) |
|  | IgA | 11 mg/dL | 50-300 mg/dL (< 3^rd^ percentile) |
|  | IgM | 8 mg/dL | 40-230 mg/dL (< 3^rd^ percentile) |
|  | Total IgE^‡^ | Undetectable (< 1 IU/mL) | < 100 IU/mL |
| IgG subclasses | |  |  |
|  | IgG1 | 150 mg/dL | 405-1,011 mg/dL |
|  | IgG2 | 31.9 mg/dL | 169-786 mg/dL |
|  | IgG3 | 59.5 mg/dL | 11-85 mg/dL |
|  | IgG4 | 1 mg/dL | 3-201 mg/dL |
| Lymphocyte subsets | |  |  |
|  | CD3 (%) | 518/µL (90.8%) | 718-2,492/µL (51.3-83.5%) |
|  | CD4 (%) | 286/µL (50.2%) | 456-1,492/µL (24.4-54.2%) |
|  | CD8 (%) | 195/µL (34.2%) | 272-1,144 µL (12.8-40.2%) |
|  | CD4/CD8 | 1.47 | 0.98-3.24 |
|  | CD19 B cells (%) | 10/µL (1.8%) | 110-618/µL (6.3-20.8%) |
|  | NK cells (%) | 31/µL (6.3%) | 82-760/µL (3.7-28.5%) |

Table E1. Laboratory parameters when patient was first seen in our Allergy and Immunology Clinics in August 2020.

^+^ Negative antinuclear and anti-thyroid antibodies, normal thyroid hormones.

^#^ Undetectable antibodies to vaccine antigens: rubella, hepatitis B, and pneumococcal polysaccharides.

^‡^ Undetectable specific IgE to a panel of food and inhalant allergens.
