# Supplementary material for: Efficacy of dupilumab for the treatment of severe skin disease in cytotoxic T lymphocyte antigen-4 insufficiency: A role of type 2 inflammation?
Source: J Allergy Clin Immunol Glob. 2022 Sep 22;2(1):114–7. doi: 10.1016/j.jacig.2022.08.004 (PMC10509893; doi:10.1016/j.jacig.2022.08.004)
Supplement: Supplementary Figure Legend [file mmc3.docx]

**Online Repository**

Figure E1. Axial CT images in lung window showing multiple pulmonary nodules (arrows), mostly solid, with irregular margins, more numerous in the lower lobes, some of them associated with ground glass opacities, as in the left lower lobe characterizing the halo sign (arrow in B). CT images obtained in July 2021 as part of evaluation following diagnosis of CTLA-4 insufficiency.
